# Revising WHO Guidelines on the management of hypoglycaemia in children

**DOI:** 10.2471/BLT.21.287416

**Published:** 2021-12-01

**Authors:** Wilson M Were, Anshu Banerjee

**Affiliations:** a Department of Maternal, Newborn, Child and Adolescent Health and Ageing, World Health Organization, Avenue Appia 20, 1211 Geneva 27, Switzerland.

In this issue, Hildenwall & Ngwalangwa highlight the challenge of clinical management of low blood glucose concentrations, which is associated with high mortality in severely sick children in low-resource settings.^1^ Hypoglycaemia is an emergency condition seen across countries of all income levels in severely sick infants and children. In low- and middle-income countries, malnutrition, high rates of infectious diseases and poor access to medical care increase the risk and severity of hypoglycaemic presentation. The diagnosis of hypoglycaemia in children is not straightforward and the definition remains controversial.^1^ Some approaches define hypoglycaemia based on symptoms, others on the plasma glucose concentration. Identifying a threshold plasma glucose concentration predictive of symptoms is difficult. The appearance of symptoms depends on potential underlying conditions and additional factors, including nutritional status and the severity and duration of hypoglycaemia.^2^

In low-resource settings, the diagnosis largely relies on surrogate clinical signs and/or a blood glucose concentration of less than or equal to 2.5 mmol/L in a well-nourished child or less than or equal to 3.0 mmol/L in a severely malnourished child. These blood glucose level cut-offs and the approach promoted by the World Health Organization (WHO) guidelines enable health workers to initiate early treatment to avoid complications.^3^ Among older children able to communicate symptoms, Whipple’s triad of symptoms consistent with hypoglycaemia,^4^ low plasma glucose concentration and resolution of symptoms with normal plasma glucose levels might be a reasonable definition.^2^

In their article, the authors suggest WHO consider increasing the definition for hypoglycaemia to a higher cut-off, using repeat glucose assessments and lowering of the 10% glucose bolus from 5 mL/kg to 2 mL/kg.^1^ However, they do not provide any evidence for the benefit and need for the higher cut-offs or harms for the current WHO 2.5 mmol/L cut-offs. In the recently published SugarFACT trial, increasing the cut-off blood glucose concentration for hypoglycaemia treatment in severely sick children in Malawi from 2.5 mmol/L to 5.0 mmol/L did not reduce all-cause in-hospital mortality.^5^ Hypoglycaemia is more of a continuum of hormonal abnormalities and clinical manifestations, and a single plasma glucose value may become difficult to associate with neurological outcome, as it could depend on the underlying condition and the degree and duration of the hypoglycaemia.^2^ In addition, an increased blood glucose cut-off will not address the challenges the authors highlight; lack of equipment, device inaccuracy, waiting time for laboratory measurements and unpredictable supply of sometimes incompatible device brands.

The authors also argue for the use of sublingual sugar mixed with water and initiating treatment in the absence of a confirmed low blood glucose concentration, which is in line with the current WHO guidelines.^6^ Children able to take oral glucose have the quickest response with higher and earlier glycaemic peak compared to sucrose.^7^ The current WHO recommendations for the management of hypoglycaemia include an initial bolus of 5 mL/kg of 10% glucose, intravenous fluids containing dextrose and oral feeds intake in addition to treatment of the underlying disease.^2^ Glucose levels should be monitored closely; however, how often monitoring should be done is unclear.^8^ For example, the American Academy of Pediatrics guidelines for neonatal hypoglycaemia noted that among infants at risk, blood glucose should be checked within 1 hour of life and 30 minutes after the feed, with continued glucose checks before feeds.^9^ Despite being a common emergency in children, controversies on the definition and management of hypoglycaemia in infants and children still exist. Neither the standard diagnostic blood glucose threshold nor the operative threshold are defined. Such uncertainties, together with the broad spectrum of causes, make the approach to hypoglycaemia in childhood complex.

Nevertheless, the authors have raised very important questions that are being addressed in the next edition of the updated WHO guidelines for management of common childhood illnesses.^3^^,^^6^ Current evidence will be reviewed on some of the key questions raised by authors – on appropriate concentration of blood glucose for diagnosing hypoglycaemia, the best choice of treatment, mode of administration and glucose therapy regimen in correcting the hypoglycaemia and improving clinical outcomes. This evidence will be presented to an independent guideline development group to decide on the appropriate recommendations; the process is expected to be completed by end of 2022.

We agree that further research on the optimal management of severely sick children who present with low blood glucose concentrations is still warranted. Many questions remain on the frequency and the clinical benefit of the repeat blood glucose measurements.

## References

[1] Hildenwall H, Ngwalangwa F. Improving management of hypoglycaemia in children. Bull World Health Organ. 2021;99(12):904–6. 10.2471/BLT.21.285586 PMC8640691 34866687

[2] Thornton PS, Stanley CA, De Leon DD, Harris D, Haymond MW, Hussain K, et al.; Pediatric Endocrine Society. Recommendations from the Pediatric Endocrine Society for Evaluation and Management of Persistent Hypoglycemia in Neonates, Infants, and Children. J Pediatr. 2015 Aug;167(2):238–45. 10.1016/j.jpeds.2015.03.057 25957977 PMC11891912

[3] Pocket book for hospital care for children: guidelines for the management of common illnesses, second edition. Geneva: World Health Organization; 2013. Available from: https://apps.who.int/iris/handle/10665/81170 [cited 2021 Oct 20]. 24006557

[4] Whipple AO, Frantz VK. Adenoma of islet cells with hyperinsulinism: a review. Ann Surg. 1935 Jun;101(6):1299–335. 10.1097/00000658-193506000-00001 17856569 PMC1390871

[5] Baker T, Ngwalangwa F, Masanjala H, Dube Q, Langton J, Marrone G, et al. Effect on mortality of increasing the cut-off blood glucose concentration for initiating hypoglycaemia treatment in severely sick children aged 1 month to 5 years in Malawi (SugarFACT): a pragmatic, randomised controlled trial. Lancet Glob Health. 2020 Dec;8(12):e1546–54. 10.1016/S2214-109X(20)30388-0 33038950

[6] Recommendations for management of common childhood conditions: evidence for technical update of pocketbook recommendations: newborn conditions, dysentery, pneumonia, oxygen use and delivery, common causes of fever, severe acute malnutrition and supportive care. Geneva: World Health Organization; 2012. Available from: https://apps.who.int/iris/bitstream/handle/10665/44774/9789241502825_eng.pdf [cited 2021 Oct 20]. 23720866

[7] Lee BM, Woleve TM. Effect of glucose, sucrose and fructose on plasma glucose and insulin responses in normal humans: comparison with white bread. Eur J Clin Nutr. 1998 Dec;52(12):924–8. 10.1038/sj.ejcn.1600666 9881888

[8] Kallem VR, Pandita A, Gupta G. Hypoglycemia: when to treat? Clin Med Insights Pediatr. 2017 Dec 15;11. 10.1177/1179556517748913 29276423 PMC5734558

[9] Adamkin DH; Committee on Fetus and Newborn. Postnatal glucose homeostasis in late-preterm and term infants. Pediatrics. 2011 Mar;127(3):575–9. 10.1542/peds.2010-3851 21357346

